# Premature expression of a muscle fibrosis axis in chronic HIV infection

**DOI:** 10.1186/2044-5040-2-10

**Published:** 2012-06-07

**Authors:** Rebecca L Kusko, Camellia Banerjee, Kimberly K Long, Ariana Darcy, Jeffrey Otis, Paola Sebastiani, Simon Melov, Mark Tarnopolsky, Shalender Bhasin, Monty Montano

**Affiliations:** 1 Boston University School of Medicine, 650 Albany St. EBRC 646, Boston, MA, 02118, USA; 2 Emory University, Atlanta, GA, 30322, USA; 3 Boston University School of Public Health, Boston, MA, 02118, USA; 4 Buck Institute, Novato, CA, 94945, USA; 5 McMaster University, Toronto, ON L8S, Canada

**Keywords:** Skeletal muscle, Aging, Gene expression, HIV infection, Senescence

## Abstract

**Background:**

Despite the success of highly active antiretroviral therapy (HAART), HIV infected individuals remain at increased risk for frailty and declines in physical function that are more often observed in older uninfected individuals. This may reflect premature or accelerated muscle aging.

**Methods:**

Skeletal muscle gene expression profiles were evaluated in three uninfected independent microarray datasets including young (19 to 29 years old), middle aged (40 to 45 years old) and older (65 to 85 years old) subjects, and a muscle dataset from HIV infected subjects (36 to 51 years old). Using Bayesian analysis, a ten gene muscle aging signature was identified that distinguished young from old uninfected muscle and included the senescence and cell cycle arrest gene p21/Cip1 (CDKN1A). This ten gene signature was then evaluated in muscle specimens from a cohort of middle aged (30 to 55 years old) HIV infected individuals. Expression of p21/Cip1 and related pathways were validated and further analyzed in a rodent model for HIV infection.

**Results:**

We identify and replicate the expression of a set of muscle aging genes that were prematurely expressed in HIV infected, but not uninfected, middle aged subjects. We validated select genes in a rodent model of chronic HIV infection. Because the signature included p21/Cip1, a cell cycle arrest gene previously associated with muscle aging and fibrosis, we explored pathways related to senescence and fibrosis. In addition to p21/Cip1, we observed HIV associated upregulation of the senescence factor p16INK4a (CDKN2A) and fibrosis associated TGFβ1, CTGF, COL1A1 and COL1A2. Fibrosis in muscle tissue was quantified based on collagen deposition and confirmed to be elevated in association with infection status. Fiber type composition was also measured and displayed a significant increase in slow twitch fibers associated with infection.

**Conclusions:**

The expression of genes associated with a muscle aging signature is prematurely upregulated in HIV infection, with a prominent role for fibrotic pathways. Based on these data, therapeutic interventions that promote muscle function and attenuate pro-fibrotic gene expression should be considered in future studies.

## Background

Despite the considerable success of highly active anti-retroviral therapy (HAART), individuals chronically infected with HIV remain at higher risk for declines in musculoskeletal function and increased frailty [1-3], a phenotype also observed in rodent models for HIV infection [4,5]. In humans, this functional decline resembles an aging phenotype, since many of these risk factors are more often seen in the elderly. However, the molecular and regulatory pathways that underlie this process remain vaguely defined.

Studies on muscle aging in both humans and rodent models have shown increases in collagen deposition and fibrotic tissue in aging muscle [6-10], suggesting a skewing in the balance of muscle and fibroblasts with increasing age. Transforming growth factor β (TGFβ) is a known key regulator in maintaining the balance of collagen in the extracellular matrix as well as in modulating inflammatory responses [11,12] . It is normally expressed in muscle after injury but also has the potential to induce fibrosis around myofibers (for review see [13]). Injection of TGFβ for ten days has been shown to induce cachexia and generalized tissue fibrosis in nude mice [14]. Furthermore, Carlson and colleagues have shown an upregulation of TGFβ and associated pathways in muscle in the aging rodent [15]. Based on these observations of muscle aging in model systems and a similar frailty phenotype in rodent models for infection, we were interested in determining whether premature muscle aging occurs during chronic HIV infection, particularly in the context of successful HAART and whether this phenotype is recapitulated in rodent models for HIV.

To evaluate potential muscle aging within the context of HIV infection, we chose to first evaluate gene expression of muscle specimens (that is, vastus lateralis) in uninfected individuals ranging in age from 19 to 85 using previously published datasets for healthy aging and mild sarcopenia to identify an aging gene signature [16,17]. To assess the possibility of accelerated muscle aging in HIV, we then evaluated muscle gene expression profiles in HIV-infected individuals ranging in age from 30 to 55 years old and in a rodent model for HIV infection and used this to identify associated pathways that might explain the phenotype seen. Senescence, a hallmark of aging, has been defined as an irreversible state of cell cycle arrest [18]. In this study, genes and pathways related to muscle senescence and fibrosis were found to be upregulated and were evaluated further. Here, we describe a role for HIV driven fibrosis in skeletal muscle. This is an understudied potential mediator of functional decline in muscle that could open novel avenues for therapeutic intervention.

## Methods

### Ethics statement

These studies were approved by the Boston University Medical Center Institutional Review Board, and the McMaster University and Hamilton Health Sciences Research Ethics Board. These studies were conducted in accordance with the principles of the Declaration of Helsinki and all subjects gave written informed consent for participation.

### Study population

#### *HIV-infected inclusion criteria*

This study included HIV-positive men, 30 to 55 years old (average age = 43 years), who had documented weight loss within the previous six months of between 5% and 15% of body weight or an actual body mass index (BMI) at screening of between 17 and 20 (equivalent to 85% to 95% of the lower limit of ideal weight), an energy intake in excess of 80% of the recommended dietary allowance, on stable and potent antiretroviral therapy for at least 12 weeks or not starting antiretroviral therapy in the next four months, CD4 cell count greater than 50/mm^3^ and HIV copy number less than 10,000 copies/ml (less than 400 in six of nine subjects), and were able and willing to provide informed consent and comply with the protocol. Expression profiles were from biopsies of the vastus lateralis at baseline that were not previously published in a testosterone supplementation study [19]. Arrays were run using Affymetrix HG-U133A microarray chips.

#### *GEO datasets*

The online datasets GEO362 [20] and GEO 1428 [16] are expression profiles of muscle tissue from normal human muscle biopsies of the vastus lateralis. GEO362 arrayed young men (age 21 to 27) and older men (age 67 to 75). GSE 1428 looked at young men (age 19 to 25) and older men (age 70 to 80) who were identified as having mild sarcopenia. The de-identified data were downloaded from the Gene Expression Omnibus (GEO) web repository and were used in our analysis. Arrays for both these studies were run using Affymetrix HG-U133A microarray chips.

### Supplemental healthy HIV negative dataset

These subjects were enrolled at McMaster University and Hamilton Health Sciences and were all healthy non-exercising males. None were athletes nor were they on medications known to adversely affect muscle (that is, statins). The age intervals for these subjects were 20 to 25 years old, 40 to 45 years old, and 70 to 75 years old. Muscle biopsies of the vastus lateralis were taken at the same time of day and fasted, as previously described [21] and profiled on Roche NimbleGen Human Gene Expression 12x135K Arrays.

### Data processing

#### *Initial processing*

All expression data were globally scaled to a per sample mean of 500. Probes were assigned to be present or absent based on the per sample median. Probes with more than 80% absent calls were removed from GSE1428 and GSE362. Only probes in GSE362 and GSE1428 with at least one sample intensity above the median for each respective set were considered for analysis using Bayesian Analysis of Differentially Expressed Genes (BADGE) [22]. As a result, a total of 9,872 probes from GSE362 and 11,016 probes from GSE1428 were included in our analysis. Remaining probes were analyzed for differential expression using BADGE [22].

### Differential expression

The GSE362 and GSE1428 data sets were analyzed independently. Our analysis found 26 upregulated and 36 downregulated genes (relative to average gene expression in the arrays being compared) for GSE362 and 19 upregulated and 65 downregulated genes for GSE1428 using a 0.5% posterior probability of false positive detection. Ten genes overlapped between the two analyses.

### Specificity assessment

To assess whether the ten gene muscle aging signature reflected a generic muscle disease phenotype, we evaluated our ten gene signature in several other muscle aging diseases such as myositis, *amyotrophic lateral sclerosis* (ALS), and 48 hour immobilization using PEPR (Public Expression Profile Resource; http://pepr.cnmcresearch.org). Our profile was only significant in 48 hour immobilization, and not any of the other diseases studied (data not shown). To test whether the ten gene muscle aging profile in HIV samples clustered selectively with the expression profiles of older subjects’ muscles, we used a Bayesian model based cluster analysis of the ten genes. The cluster method was implemented in the software CAGED (Cluster Analysis of Gene Expression Dynamics) which was also used to generate the unsupervised clustered heatmaps [23].

### EASE annotation

Annotation of our gene lists was performed using the National Center for Biotechnology Information (NCBI) software EASE [24]. EASE is an integrated knowledge database that integrates information from OMIM, Refseq, Unigene, and Gene Ontology to search for overrepresented gene categories in user submitted gene lists [25].

### Ex vivo validation and pathway analysis

#### *TGFβ protein measurement*

Muscle homogenates were obtained from the gastrocnemius muscle of the wild type and HIV Tg rodent. Briefly, samples were cleaned of all fat and connective tissue and cut into smaller pieces. Specimens were homogenized in RIPA buffer (25 mM Tris pH7.6, 150 mM NaCl, 1% NP-40, 1% sodium deoxycholate, 0.1% SDS) with complete mini protease inhibitor tablets added (Roche, IN, USA). Homogenates were centrifuged at 21,000 g for 15 minutes to pellet insoluble matter. Protein concentrations were determined using the BCA reagent (Pierce, Rockford IL, USA). TGFβ was measured by ELISA using a Quantikine kit (R&D Systems, Minneapolis, MN, USA) according to the manufacturer’s recommendations and values were normalized to total protein concentration. Three HIV Tg and three wild type rodents were used for these measurements. A Students *t*-test of the data was used to test differential expression with a *P*-value < 0.05 considered as significant.

### RNA quantitative realtime PCR

RNA was generated from gastrocnemius muscles. Briefly, the gastrocnemius muscles were cleaned of all fat and excess connective tissue, cut into smaller pieces and homogenized in TRIzol reagent (Invitrogen, Carlsbad, CA, USA}) then processed for RNA. Phase separation was done using chloroform and centrifugation. RNA was precipitated from the aqueous phase using isopropynol and washed using ethanol. The RNA was then cleaned using the RNeasy Mini Kit (Qiagen Sciences, Valencia, CA, USA) and quantified using a Nanodrop spectrophotometer. Primers for rat GAPDH, CDKN1A, FEZ2 and H3F3B were purchased from Superarray (SABiosciences, Frederick, MD, USA) and analyzed using quantitative realtime PCR detection with SYBR green. For genes associated with fibrosis, COL1A1 (F-GGAATGAAGGGACACAGAGGT, R- GAGCTCCATTTTCACCAGGA), COL1A2 (F-GAGCTCCATTTTCACCAGGA, R- CAGCAGCTCCACTCTCACCT), CTGF (F-ATGCTGTGAGGAGTGGGTGT, R- GGCCAAATGTGTCTTCCAGT) and GAPDH (F- ATGACTCTACCCACGGCAAG, R- GGAAGATGGTGATGGGTTTC), primers were used - a kind gift from Dr. Maria Trojanowska (Boston University, MA). They were analyzed using quantitative realtime PCR detection using SYBR green. TaqMan probes for rat p16INK4a, MT1A, MLF1, TPPP3 (also known as CGI-38), MYH8, PDHA1 and GAPDH were purchased from Applied Biosystems (Life Technologies, Carlsbad, CA, USA). Both sets were analyzed using an ABI Prism 7000 Sequence Detection System (Applied Biosystems, Life Technologies, CA, USA). Samples were confirmed to have no DNA contamination by using a realtime PCR reaction without reverse transcriptase. Amplification results were normalized to GAPDH using the ΔΔCt method. A Students *t*-test of the data was used to test differential expression with a *P*-value < 0.05 considered as significant.

### Collagen staining and fibrotic index calculation

Gastrocnemius muscle was flash frozen in liquid nitrogen and later embedded in paraffin. Sections of tissue were processed at 5 μm thickness. Tissue was stained using Picrosirius Red (Sigma-Aldrich, St. Louis, MO, USA}) and Fast Green (Fisher Scientific, Hampton, NH, USA) to look for collagen deposition in the extracellular space. The images for analysis of fibrotic index were taken using Olympus BX41 microscope (Olympus, Center Valley, PA, USA) using DP Controller (Version 3.2.1.276) and DP Manager (Version 3,1,1,208). Bright field images were exposed for 1/1500 seconds. The fibrotic index was calculated as percent area of collagen of the total tissue area using NIH Image J Software (http://rsbweb.nih.gov/ij/). The calculation for fibrotic index is based on the algorithm described in [26]. Six HIV Tg and three wild type rodent muscles were quantified after staining. A Students *t*-test of the data was used to test differential expression with a *P*-value < 0.05 considered as significant.

### Myosin heavy chain isoform quantification

Gastrocnemius muscles were embedded in OCT and immediately frozen in isopentane cooled in liquid nitrogen. Serial sections from the mid-belly of the gastrocnemius were cut at 8 μm and processed for immunohistochemical detection of slow or fast MHC protein expression using the ABC method (Vector Labs, Burlingame, CA, USA). Sections were visualized with a Leica microscope and measured using ImageJ software (NIH, Bethesda, MD, USA). Approximately 200 fibers per muscle were analyzed. Data are expressed as the percentage of slow (type I) and fast (type II) MHC types relative to the total pool of MHC isoforms. Four HIV Tg and four wild type rodent muscles were quantified. A Students *t*-test was used to test differential expression with a *P*-value of less than 0.05 considered as significant.

## Results and discussion

### Identification of common genes that change expression with muscle aging

**Figure 1:**
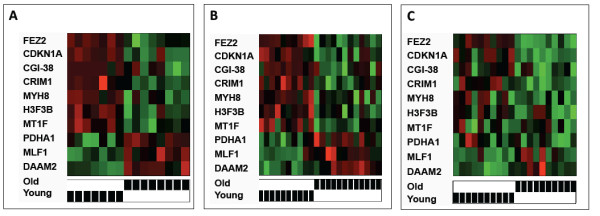
**Muscle age profiling.** Heatmaps displaying the relative expression of the ten gene muscle aging profile are shown comparing young males to older males in a supervised manner using Affymetrix U133A microarray gene set data for GSE362 (**A**) and GSE1428 (**B**) and in a healthy aging dataset using a NimbleGen microarray data (**C**). The gene expression of the profile is displayed in a supervised manner where intensity reflects relative expression (green = higher, red = lower)**.** Heatmaps were generated using the Heatplus package in the statistical software R 2.14.0

To profile muscle in healthy aging, we identified a shared gene expression pattern in healthy and mildly sarcopenic individuals using previously published expression data obtained from the same tissue and microarray platform (that is, Affymetrix HG-U133A). We then evaluated this expression pattern with microarray data from our HIV muscle specimens using the same microarray platform (that is, Affymetrix HG-U133A) as well as with a distinct microarray dataset of young, middle aged and older men using a NimbleGen platform (Table 1). A Bayesian modeling approach was applied, implemented in BADGE [22,23], using muscle expression datasets obtained from the vastus lateralis in GEO series 362 (a dataset including seven young healthy men ages 21 to 27 and eight older men ages 67 to 75 [20]) and GEO series 1428 (a dataset including ten young healthy men ages 19 to 25 and twelve older men with mild sarcopenia ages 70 to 80 [16]). The initial analysis identified 62 age-associated probes that were differentially expressed in the dataset GSE362 and 85 age-associated probes differentially expressed in the dataset GSE1428. Ten genes were common to both expression sets and are shown for the two datasets as heatmaps in Figures 1A and B. Notably the mild sarcopenia in the GEO 1428 series displayed a larger number of differentially expressed genes compared to healthy subjects (data not shown). This ten gene signature was evaluated in another supplemental healthy male dataset run on a NimbleGen microarray platform, comparing subjects 20 to 25 years old with subjects 70 to 75 years old. Most genes (the top six of ten in the heatmap) in the signature were recapitulated in the third set (Figure 1C). The composition of the ten genes shown in all three datasets is as follows: CDKN1A (p21/Cip1), FEZ2, H3F3B DAAM2, MFL1, PDHA1 MT1F, MYH8, CRIM1, and CGI-38.

**Table 1:** Description of datasets used in this study

**Dataset**	**Age(yrs)**	**Sample Size(all male)**	**Tissue source**	**Array**	**Condition**
GSE362 [20]	21-2767-75	N = 7N = 8	Vastus lateralis	HG-U133A	Healthy
GSE1428 [16]	19-2570-80	N = 10N = 12	Vastus lateralis	HG-U133A	Older with sarcopenia
HIV + [19]	36-51	N = 9	Vastus lateralis	HG-U133A	Median VL = 400 copies/ML (400–56,844)Median CD4 = 362 cells/CMM (61–765)
Supplemental Healthy	20-2540–4570-75	N = 10N = 10N = 10	Vastus lateralis	NimbleGen	Healthy

The GSE362 and GSE1428 genome-wide expression data have been previously published and are available online [16,20]. The HIV baseline muscle profiles and the supplemental healthy muscle expression profiles have not been previously published. Cells/CMM = cells per cubic milliliter.

### Muscle gene expression of middle-aged HIV-infected men resembles older uninfected samples

**Figure 2:**
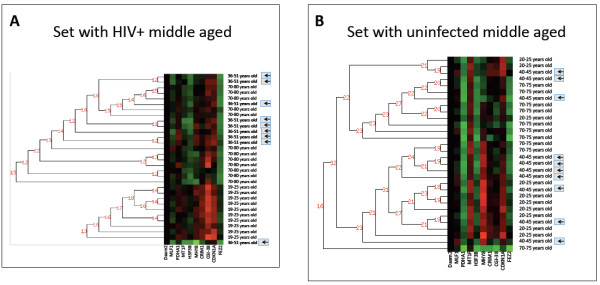
**A. Muscle age profiling with HIV.** Heatmap displaying the ten gene profile shown in Figure 1, with the HIV group added using GSE1428 (Figure 2A) and GSE362 (similar results, data not shown). The CAGED software was used to cluster samples (shown as rows of the heat map) based on the ten gene muscle aging profile (columns of the heat map). The analysis shows that the young men form a cluster, while HIV samples (designated by arrows and in bold, 36 to 51 years old) cluster with old subjects using the ten gene muscle aging profile. The CAGED cluster analysis uses a model-based procedure that assigns samples to the same cluster if their merging increases the posterior probability of the model. Numbers attached to the branches of the dendrogram represent the posterior odds of the model that merges the branches versus the model that does not. **B.** CAGED analysis of the expression of the ten gene muscle aging profile is an HIV negative dataset with young (20 to 25 years old), intermediate (40 to 45 years old designated by arrows), and old (70 to 75 years old) subjects (this entire dataset used the microarray NimbleGen platform). The gene expression of the profile is displayed in a supervised manner where intensity reflects relative expression (green = higher than average, red = lower than average). CAGED, Cluster Analysis of Gene Expression Dynamics

Using the muscle aging profile common to both datasets, we evaluated muscle expression profiles from HIV-infected individuals using the same tissue source (vastus lateralis) and microarray platform (Affymetrix HG-U133A). The samples are described in [19]. Muscle profiles from these subjects clustered with the older samples in both GSE362 (shown in Figure 2A) and GSE1428 (data not shown) using CAGED [23] cluster analysis. Notably, the clustering was not influenced by viral load, extent of weight loss or CD4 levels (data not shown). Collectively, these data suggest that muscle derived from HIV + men, on average in their 40s, more closely resembles muscle profiles from individuals in their 70s consistent with our premise of accelerated muscle aging.

### Muscle gene expression of age-matched healthy HIV negative men do not resemble older samples

We evaluated muscle profiles from young, middle aged and older uninfected men, all derived using the same tissue site and microarray platform (Table 1), to determine whether age, rather than HIV, might account for the observed clustering in Figure 2A. However, CAGED cluster analysis of all three groups showed the middle aged men clustering randomly with the young group and the old group, see Figure 2B. Notably, when middle aged healthy men were removed from the analysis, we again observed clear partitioning of the young from the old (Figure 1C), confirming that young and old in this dataset also recapitulate the ten gene muscle signature, as observed in Figure 1 A and B. These data are consistent with the possibility that HIV infection promotes premature expression of this gene signature in muscle.

### Expression of the aging signature in a transgenic rodent model for chronic HIV infection

**Figure 3:**
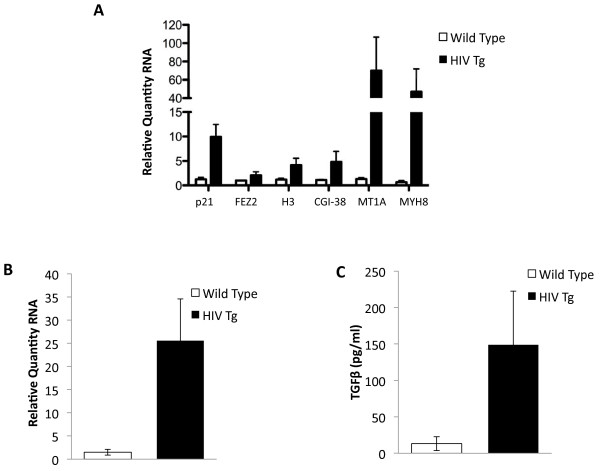
**Expression of p21/Cip1, p16INK4a and TGFβ1 in the HIV transgenic rodent. A**. Realtime RNA PCR validation was observed for p21/Cip1, Fez2, H3, MFL1, MT1F and MYH8 in muscle from HIV transgenic gastrocnemius (n = 4) muscle or wild type gastrocnemius muscle (n = 3) and shows an increase in genes in HIV Tg compared to age matched wild type controls. All bar plots show mean fold change with error bars indicating the standard deviation. The difference between wild type and HIV Tg is significant to *P* ≤ 0.05. **B**. RT-PCR shows an increase in the cell cycle arrest gene, p16INK4a, in HIV Tg rat (n =4) compared to wild type (n = 3). The difference is significant to *P* < 0.05. The bar plot indicates mean fold change with the error bars indicative of the standard deviation. **C**. TGFβ1 protein levels based on ELISA of muscle homogenates from HIV transgenic gastrocnemius muscle (n = 3) or wild type gastrocnemius muscle (n = 3) show increased levels in the HIV Tg rodent at a significance of *P* < 0.05. Bar plots indicate mean protein levels with error bars indicative of the standard deviation. TGFβ, transforming growth factor β

One of the genes in our signature, the cyclin dependent kinase inhibitor (CDKi) p21/Cip1, has been associated with muscle aging in rodent models for aging [6,15]. We were, therefore, interested in whether a transgenic rodent model for HIV infection (HIV Tg) would display premature elevation of p21/Cip1 expression as well as the other genes in our signature. The HIV Tg rodent expresses a transgene consisting of a HIV provirus with a functional deletion of *pol* and *gag* regulated by the viral long terminal repeat. These rodents share many similarities to human HIV infection, compared to other rodent models. Specifically, these rodents express the virus in lymph nodes, spleen, kidney, thymus and immune cells including macrophages, T cells and B cells, are antigenic to gp120 and shed gp120 into the peripheral blood stream and have immune suppression compared to wild type animals. Furthermore, by five to nine months of age, these animals develop weight loss, neurological abnormalities, respiratory difficulties and other symptoms of AIDS [27,28]. We chose to use the HIV Tg rodent model for chronic infection because this model displays musculoskeletal decline that includes loss in lean muscle and resorption of bone, both phenotypes observed in human HIV infection [4,5]. As shown in Figure 3A, we observed significant up-regulated expression of p21/Cip1, as well as most of the other aging signature genes or gene homologues (for example, Fez2, H3F3b(H3), CGI-38, MT1, MYH8) using quantitative real time PCR analysis in concordance with expression in the microarray profiles observed in our human muscle specimens. CRIM1 was found to show a trend increase with the HIV Tg compared to the wild type but did not show statistical significance (*P* = 0.08). Notably, three genes, PDHA, DAAM2 and MLF1 were not significantly different between the wild type and HIV transgenic rat (data not shown), possibly indicating species-specific regulation.

### p16INK4a is upregulated in a transgenic rodent model for chronic HIV infection

Recent data have linked elevated p21/Cip1 to other cell cycle arrest genes such as p16INK4a, with healthy aging in muscle stem cells [15] and other tissue specific stem cells [29,30]. To assess whether cell cycle arrest was a general feature in the muscle in our rodent model, we measured p16INK4a RNA levels in both the HIV Tg and wild type rodent. Figure 3B shows that the levels of p16INK4a were significantly elevated compared with wild type age-matched controls. This further suggests that cell cycle arrest genes are prematurely expressed in muscle during HIV infection, specifically that both the Cip and Ink4 families are induced.

### TGFβ is upregulated in a transgenic rodent model for chronic HIV infection

The TGFβ family members, including TGFβ itself, as well as the growth antagonist myostatin, have been shown to upregulate p21/Cip1 and p16INK4a in aging muscle in both the stem cell population and the muscle fibers in mice [6,15]. Expression of both p21/Cip1 and p16INK4a has also been shown to be upregulated in a number of human aging studies in leukocytes, fibroblasts, neural tissue and pancreatic islet cells [18,29-39]. This upregulation of p16INK4a is thought to be associated with aging and senescence. To evaluate whether TGFβ protein might also be upregulated in our HIV muscle in addition to the senescence-associated genes, we measured protein levels in muscle homogenates from HIV Tg and age-matched wild type rodents at the onset of bone and muscle loss (approximately seven months of age). As shown in Figure 3C, we observed a dramatic increase in TGFβ protein levels in HIV Tg animals compared to wild type controls, consistent with elevated TGFβ in muscle aging studies in rodents [15].

### The collagen gene inducer, CTGF, as well as collagen gene expression are induced in a transgenic rodent model for chronic HIV infection

**Figure 4:**
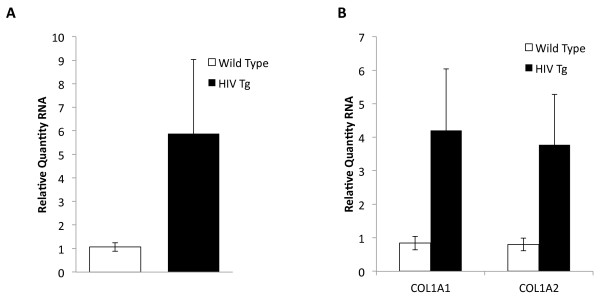
**Expression of collagen genes in the HIV transgenic rodent model. A.** The collagen transcriptional regulator, CTGF, is increased in HIV Tg rat muscle (n = 3) compared to wild type (n = 3) using realtime RNA PCR at a significance of *P* <0.05. The bar plots indicate mean fold change with error bars indicative of standard deviation. **B.** The collagen genes, COL1A1 and COL1A2 are increase in HIV Tg rat muscle (n =3) compared to wild type (n = 3) using realtime RNA PCR. The bar plots indicate mean fold change with the error bars indicative of standard deviation. The difference between wild type and HIV Tg rodents are significant to *P* < 0.05. CTGF, connective tissue growth factor

Elevated TGFβ levels have been previously linked to deposition of collagen and tissue fibrosis of lymph nodes in both human HIV infection and in simian immunodeficiency virus (SIV) models of infection [40,41]. Furthermore, in aging, muscle from rodents has been shown to convert to a fibrogenic phenotype [6]. However, fibrotic genes and collagen deposition in skeletal muscle have not yet been described in the context of HIV infection. We, therefore, measured genes associated with TGFβ and collagen deposition in the hindlimb muscle (gastrocnemius) of the wild type and HIV Tg rodent. We first examined the collagen transcriptional inducer, connective tissue growth factor (CTGF), a factor that mediates TGFβ induced collagen gene expression [42,43]. As shown in Figure 4A, there was an increase in expression in CTGF in the HIV Tg rodents. We then measured the expression of collagen genes, COL1A1 and COL1A2. As shown in Figure 4B, these genes were also upregulated in the HIV Tg rodent.

### The HIV tg rodent muscle exhibits fibrosis and fiber type switching in muscle

**Figure 5:**
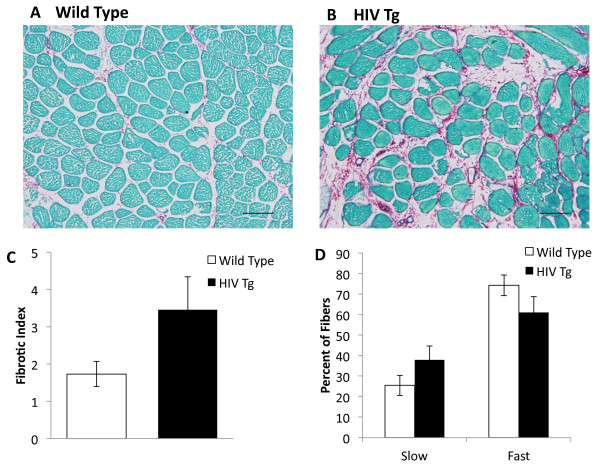
**Collagen deposition and fiber type switching in the HIV transgenic rat.** Histological muscle sections of the gastrocnemius muscle of HIV Tg (n = 6) and control wild type rats (n = 3) were stained using Picrosirius Red for collagen. Representative images are shown in **A** and **B**. The scale bar equals 200 μm. **A.** Tissue section of the gastrocnemius muscle in wild type rat shows collagen deposition in red in intracellular space. **B.** Tissue section of gastrocnemius muscle from HIV Tg rat shows increased collagen deposition in red in the intracellular spaces. **C.** Quantification of the area of picrosirius red staining using Image J shows that there is an increase in fibrotic index in the HIV Tg rat compared to the wild type at a significance of *P* < 0.05. The bar plot indicates mean percent area of collagen with the error bars indicative of the standard deviation. **D.** Quantification of fiber type was done using Image J on control wild type (n = 4) and HIV Tg (n = 4). The bar plot indicates the percentage of fibers of each type with error bars indicative of standard deviation

Because we saw evidence of the upregulation of collagen genes in HIV, to evaluate whether collagen deposition was detectable in association with upregulated TGFβ and collagen genes, we analyzed gastrocnemius tissue sections and quantified collagen content. As shown in Figure 5, there was a significant increase in muscle collagen deposition in the HIV Tg rodent compared to the wild type controls. This is consistent with aging muscle, which also shows an increase in collagen deposition [6]. Since aging is also associated with a shift in fiber types, specifically an increase in slow twitch and a decrease in fast twitch [44-46], we analyzed the types of fibers seen in the gastrocnemius in the wild type compared to the HIV Tg. We found that as with aging, there is a significant increase in slow twitch fibers and decrease in the fast twitch fibers in the HIV Tg rat compared to the wild type.

## Conclusions

With the success of anti-retroviral therapy in suppressing viral burden there has been a profound influence on the course of HIV infection from a lethal to a managed chronic disease. Nevertheless, HIV-infected individuals remain at higher than expected risk for a number of complications typically associated with aging [47]. Aging is often defined on the basis of functional capacity, or frailty, rather than the collection of age-associated diseases [48,49]. Frailty, as defined in the elderly by Fried and colleagues, [50] has been previously evaluated in men with HIV [1,2] wherein a significant frailty-related phenotype (FRP) has been observed. Margolick and colleagues have also reported the premature occurrence of age-adjusted frailty with HIV infection [1-3].

With the advancing age of the HIV-infected population, the role of aging-associated inflammation in compromising normal tissue remodeling is becoming increasingly more relevant. In 2006, approximately one third of people with AIDS in the US were over age 50 [49]. Chronic inflammation that occurs with increasing age has been shown to adversely affect muscle homeostasis [51-53], with aging muscle increasingly likely to exhibit changes in extracellular matrix resembling a fibrogenic phenotype [6-9]. Recently, SIV infection has been linked with fibroblast deposition of collagen and increased fibrosis in lymph nodes associated with an increase in local TGFβ production [40,54,55]. However, a detailed study of fibrosis in skeletal muscle in chronic HIV infection has not been done to date. In this study, we use an aging signature in muscle to identify and evaluate aging- and fibrosis-related pathways in the rodent model of HIV. We show gene expression changes and phenotypic changes in muscle in an HIV Tg rodent model similar to that seen in aging. We see an upregulation of genes associated with aging muscle prematurely expressed in HIV in both the human and the rat. We, furthermore, show changes in both fiber type composition and in fibrotic collagen deposition in the HIV rodent. Previous work by our laboratory in humans has also shown that an increase in CTGF expression in vastus lateralis from HIV-positive men was downregulated with anabolic supplementation in microarray analysis (see supplemental data in [19]), consistent with our findings of a dysregulation of CTGF in HIV. Collectively these data suggest that the genotypic and phenotypic changes that we see might be related to HIV infection. This data also elucidate pathways HIV might use that result in increased risk for a frailty-related phenotype.

Three independent datasets were used in this study to identify an aging gene signature that differs between older individuals and younger individuals. This signature is prematurely expressed in HIV-positive subjects. The aging profile genes include: CDKN1A/p21, FEZ2, H3F3B, DAAM2, MLF1, PDHA1, MT1F, MYH8 and CRIM1. FEZ2 is a factor that interacts with a number of transcription controlling proteins to influence chromatin remodeling and apoptosis [56]. H3F3B is a replacement histone that constitutes the major form of histone H3 in senescent cells [57]. DAAM2 regulates cell fate and actin-cytoskeleton re-organization through WNT signaling [58]. Previous studies have shown that changes in actin cytoskeleton turnover can trigger an increase in levels of reactive oxygen species (ROS) in the cytosol and, therefore, decrease cellular viability [59]. MLF1 suppresses COP1 through COP9 thereby stabilizing p53 [60]. PDHA1 is a mitochondrial metabolic enzyme that provides the link between glycolysis and the TCA cycle [61]. MT1F and MT1A have a metalloregulatory function in repair, growth, and differentiation [62] with MT1A being a similar family methallothionein to MT1F in the rat. MYH8 is a member of the myosin protein family and is involved in muscle contraction by interacting with actin filaments [63]. CRIM1 is a membrane bound protein that is known to interact with the TGFβ superfamily [64]. CDKN1A (p21/Cip1) is a well-recognized cell cyclin dependent kinase inhibitor that has been associated with muscle aging and cellular senescence that has not been previously linked with muscle aging in HIV infection. Whether these genes reflect a larger network of regulators in aging that are co-opted in HIV is beyond the scope of this study and will require further network and pathway analysis of these genes and their potential interactions during healthy and dysregulated muscle aging. It is of note that while these genes displayed a robust signature in our two initial datasets, GSE363 and GSE 1428, the signature did not fully recapitulate in a third cohort (shown in Figure 1C). Furthermore, the genes that erode in the heatmap for the third cohort include PDHA, MLF1 and DAAM2 and are the same genes that did not show significant changes in the HIV Tg rat model compared to the wild type (data not shown). This data suggest that these three genes may not remain as robust as the others and will require further analysis of additional independent datasets.

In this study, we see selected senescence-ssociated pathways in addition to the fibrosis pathways prematurely expressed in HIV infection. Based on these results, we propose that HIV infection may represent an uncoupled and dysregulated aging phenotype that increases the risk for muscle fibrosis. Senescence-associated pathways have been previously implicated in muscle aging, but have not been directly linked to HIV infection. Recent studies on senescence and aging indicate that cellular senescence is accompanied by a striking increase in the secretion of 40 to 80 factors [34,38,65,66], termed the ‘senescence-associated secretory phenotype’ or SASP [35], an ensemble of factors that includes multiple inflammatory cytokines. Previous studies on senescence show that the cells responsible for the release of SASP factors show an upregulation in key senescence-associated genes including p21 and p16INK4a [35-37,39,65]. In muscle, these genes are thought to contribute to a decreased regenerative capacity with aging that has been seen in rodents as they have been detected in both fibers and satellite cells [15,53]. We show the premature upregulation of p21/Cip1 in both human and rodent muscle tissue with HIV and the premature upregulation of p16INK4a in rodent muscle tissue suggesting that a senescence axis might be activated early with HIV infection. What cell types in the muscle (that is, satellite cells, fibroblast or mature muscle cells) contribute to the changes in gene expression is beyond the scope of this study and will require further analysis. Furthermore, whether or not the upregulation of these genes contributes to a dysregulation of the satellite cells in HIV muscle leading to an aging phenotype is an intriguing area of future study.

As previously mentioned, many of the factors that increase in serum with aging include inflammatory cytokines including TGFβ, TNFα and IL-6 [34-36,38,39,67-69]. Some of these circulating factors have been shown to dampen proliferation of satellite cells, lead to remodeling of the stem cell niche and lead to dysregulation of stem cell function specifically in muscle [15,53,70]. HIV is also associated with an increase in systemic inflammatory burden, especially in serum factors like IL-6 [1,2,69,71,72]. Increases in systemic inflammatory cytokines are also known to lead to a loss of muscle mass [73-78]. Whether the gene signature that we see in HIV and aging is due to a common increase in inflammatory burden or whether HIV infection itself drives both the signature and inflammatory process is an intriguing question that remains beyond the scope of this study.

In addition to inflammation, previous studies dissecting acute conditions leading to muscle atrophy have revealed a common role for the ubiquitin proteasome pathway mediated by the E3 ligases MuRF1 and MAFBx to promote muscle protein degradation, notably the muscle structural protein myosin heavy chain [79-82]. Upstream activators for these atrogenes include FOXO and NFκB pathways [83] and have been identified in multiple rodent models of induced wasting, notably cancer-associated cachexia [84] and also HIV in a rodent model for infection [5]. While we do not see an upregulation of atrogenes in our genetic signature in aging, the HIV transgenic rodent model has previously been shown to have an increase in MuRF1 compared to the wild type [4]. Therefore, the atrogene pathway may represent another pathway in addition to the fibrotic and senescence pathways we study that shows dysregulation from the normal aging pattern seen specifically in HIV.

The cause of HIV-related frailty and muscle loss is an area that has been understudied with the etiology poorly understood. Further evaluation of both the senescent and fibrotic pathways and genes in association with muscle function in healthy aging and in HIV infection should better assist in identifying molecular pathways associated with frailty in both conditions. Increased frailty is likely to create a substantial burden on future health care, underscoring a need for diagnostic biomarkers and early detection in this population. The role of chronic inflammation driven fibrosis in HIV is an understudied potential mediator of functional decline. Further characterization of this phenotype could open avenues for therapeutic intervention to prevent fibrosis associated declines in physical function.

## Abbreviations

AIDS: Acquired Immunodeficiency Syndrome; ALS: amyotrophic lateral sclerosis; BADGE: Bayesian analysis of differential gene expression; CAGED: cluster analysis of gene expression dynamics; CDKi: cyclin dependent kinase inhibitor; CMM: cubic millimeter; CTGF: connective tissue growth factor; ELISA: enzyme-linked immunoassay; FRP: frailty related phenotype; GEO: gene expression omnibus; HAART: highly active antiretroviral therapy; HIV Tg: HIV transgenic; HIV: human immunodeficiency virus; IL-6: interleukin 6; MAFBx: muscle atrophy F-box; MHC: myosin heavy chain; MuRF1: muscle RING finger protein 1; NCBI: National Center for Biotechnology Information; OCT: optimal cutting temperature medium; OMIM: Online Mendelian Inheritance in Man; PCR: polymerase chain reaction; RNA: ribonucleic acid; ROS: reactive oxygen species; SASP: senescence associated secretory phenotype; SIV: simian immunodeficiency virus; TCA: tricarboxylic acid cycle; TGFβ1: transforming growth factor β 1; TNFα: tumor necrosis factor α; US: United States; WT: wild type.

## Competing interests

The authors declare that they have no competing interests.

## Authors’ contributions

BK and CB contributed equally to this work. BK, CB and MM wrote the manuscript. KL, CB and AD performed the experiments. JO, SM, MT and SB provided muscle tissue and/or unpublished microarray expression data. BK, PS and MM conceived of the study, coordinated the experimental approach and drafted the manuscript. All authors read and approved the final manuscript.
